# In the eye and mind of the beholder: The effects of familiarisation on the perception of atypical infant facial configurations

**DOI:** 10.1371/journal.pone.0311763

**Published:** 2024-11-12

**Authors:** Benjamin W. Hunt, Leonardo De Pascalis

**Affiliations:** 1 Department of Psychology, University of Liverpool, Liverpool, United Kingdom; 2 Department of Psychology, University of Bologna, Bologna, Italy; Universiti Malaya Fakulti Perubatan: University of Malaya Faculty of Medicine, MALAYSIA

## Abstract

Perception of infant faces plays a crucial role in adult-infant caretaking behaviour, with adults being found to demonstrate a reliable attraction towards infant faces over other stimuli. When affected by a congenital facial malformation such as cleft lip and/or palate, however, adults’ visual scanning patterns and subjective appraisal of these faces have been found to be adversely affected. Little past work has explored how an observer’s prior experience with this specific malformation might play a role in the perception of cleft-affected infant faces. To this end, two groups of adult female participants were recruited and presented with 48 images of infant faces (24 typical, 24 cleft-affected) with one group subjected to novel, purpose-built familiarisation training, where participants were exposed to infant cleft lip/palate related visual and informational stimuli prior to testing (*n* = 43). Eye gaze patterns and subjective “cuteness” ratings from this group were compared with an age matched control group which received no training (*n* = 41). No between group differences were found for “cuteness” ratings or eye gaze patterns to the cleft-affected mouth area in isolation, however, a significant negative relationship was found between gaze duration to the mouth region of cleft-affected images and subjective “cuteness” ratings, for control participants only. Notably, this relationship was not observed for the familiarised participants, suggesting their modified prior experience attenuated the effect that visual processing had on subjective appraisal of cleft-affected faces, when these two factors were assessed in tandem. Our findings suggest it is possible to attenuate the typically observed aversive behaviour towards cleft-affected infant faces. This may have implications for clinical practice concerned with supporting adult caretaking of malformation-affected infants and policies related to increasing positive perception of congenital facial disfigurement.

## Introduction

When presented with the opportunity, adult humans prefer infant faces over adult faces [1], demonstrated by the tendency for adults to provide higher subjective ratings on indices such as “likeability” and “attractiveness” for infant compared to adult faces [2]. Adults’ attraction to infant faces has been found to be related to the facial features typical of infants and young children [3]. This arrangement of facial features has been termed “Kindchenschema” or baby schema [4] and refers to the specific structure and characteristics of the infant, typically present in the face, such as a large head and eyes [3], with infants higher on the baby schema spectrum having more pronounced features (e.g., bigger eyes, chubbier cheeks) compared to infants lower on this spectrum [5].

Adults tend to provide higher subjective attractiveness ratings of infants the higher the presence of baby schema features [5–7]. In addition, younger infants tend to receive higher attractiveness ratings than older infants [8–10], which suggests infant attractiveness has an evolutionary role, likely related to the infant’s vulnerability, geared toward the adult’s care and protection of the infant.

These infant directed responses are likely influenced by differences, not only among infants, but also relating to the adults involved in them. Studies have found differences among adult observers to influence how infant faces are perceived, with sex of the observer being one such example. Females tend to show more interest in interacting with infants [11], work harder to view infant faces (i.e., by expending more effort in a key press paradigm) [12] and tend to provide higher subjective attractiveness ratings [5] than males. Additionally, age of the female observer has been found to be related to subjective “cuteness” ratings of infant faces [11] with younger females providing higher ratings and being more sensitive to experimentally manipulated differences in infant “cuteness” [13] compared to older females.

Perceived infant “cuteness” can also be influenced by congenital factors relating to the infant’s appearance. Experimental research has found that, compared to typical infants, adults provide lower subjective ratings (e.g., “cuteness”, attractiveness) [14, 15], ascribe more negative emotional valence [16], and display reduced motivation to view images of infants affected by a facial abnormality during key press tasks [17, 18]. This reduced preference and motivation to view infants with a facial malformation has been suggested as representing an overt behavioural response to the disrupted baby schema [15].

A population that appears to hold special interest for scientific investigation is that of infants affected by cleft lip and/ or palate (CLP), as previous research has found that the structural alteration entailed by the cleft alters how the face is encoded by the brain during face processing [19]. Therefore, CLP provides the opportunity to investigate adult responses to infant faces where the baby schema has been disrupted in a naturalistic setting. Indeed, the wealth of previous research that has focused on adult responses towards cleft-affected infant faces is likely related to its relatively high incidence rate for congenital facial malformations (1 in 700 in the UK as of 2018; [20]) and its value in exploring the processing of infant faces in the presence of natural alteration to the baby schema.

One experimental method that is particularly suitable for exploring human responses to faces is eye tracking, which has the advantage of objectively capturing the location of an individual’s point of fixation and amount of time spent viewing target stimuli in a controlled environment [21]. Eye tracking methods have been employed in an attempt to explore observers’ perceptual processes [22], attention to salient stimuli [23], and decision making [24]. Research has found a positive relationship between gaze dwell time towards a target and the degree to which the target contains unpleasant or dysphoric aspects [23], suggesting participants show a bias towards targets that contain these characteristics.

Similarly, research has employed eye tracking towards face images, in an attempt to elucidate the visual processes involved in face perception. For example, when presented with images of individuals varying in age, participants dwell for significantly longer on older compared to younger adult faces [25], and, with regards to facial regions of interest, participants show a tendency to gaze towards the eyes and frequently revisit the eye region during free viewing tasks [26].

Relatedly, a wealth of research has now employed eye tracking methods in a range of studies that have investigated adult gaze behaviour toward both typical and atypical infant populations [15, 27–29]. Previous research on adult gaze to infant faces has found that, when presented with images of cleft-affected infants, naïve observers demonstrate a remarkable tendency to disproportionately fixate their gaze on the affected infants’ mouth area, at the expense of reduced fixation to the eyes [15, 30], with a stronger gaze to mouth bias being found for more severe clefts [15]. Studies using eye tracking often employ a measure of subjective appraisal towards the target image in tandem with the collection of gaze data [15, 31, 32], to explore the relationship between the two measures. Boonipat et al. [33] found naïve participants spent more time gazing at the upper lip region of cleft-affected faces rated as less attractive. Relatedly, Rayson et al. [15] reported a positive association between participants’ viewing time to the eye area of CLP-affected infant images (with reduced gaze time to the mouth occurring as a consequence) and “cuteness ratings”. Based on these previous findings, it may be reasonable to expect gaze to the mouth area and subjective “cuteness” ratings to be inversely related, due to the presence of the cleft and subsequent violation of the baby schema [4], and because of the generally aversive nature of the malformation [18, 34].

Given the naïve observer’s inherent lack of familiarity with CLP, and the relative novelty this facial malformation represents for them, it may initially seem unsurprising that naïve participants display a tendency to focus on the mouth region of infants affected by CLP. This behaviour is not however shared by all populations of potential observers. When mothers who have given birth to an infant with CLP have been observed interacting with their cleft-affected infant, they showed remarkably different behaviour, focusing their visual attention on facial areas other than the mouth, in the first weeks of the infants’ lives [28].

It would be reasonable to view this difference in responses between the two groups as being related to the several factors that differentiate a naïve participant observing a static image of an infant face with a cleft lip, from a mother interacting in real time with her infant with the same kind of congenital facial malformation. Aside from the notable difference in stimulus presentation, the latter group of individuals possesses several ‘nested’ characteristics known to affect the processing of infant related stimuli: they are mothers of those specific infants they are observing [35–38], mothers of cleft-affected infants [39], and mothers in general [40–42]. While recognising these group differences, and their probable role in the aforementioned difference in findings on naive observers *vs*. mothers, we aim to focus on the "lowest common denominator" characteristic shared by the two groups, the simplest possible common aspect of their visual processing experience, that of being adult observers gazing at an infant face with a cleft lip. One factor that sits within this common characteristic, but which differs in degree between the two groups, thus potentially contributing to their different processing of cleft-affected infant faces, is the level of perceiver familiarity with infant CLP.

Mothers of infants born with a cleft become familiar with CLP related stimuli in a number of ways. Firstly, once the infant has been delivered, these mothers are being routinely exposed to their infant’s malformation during typical mother-infant interaction. However, it is plausible that some familiarity has been established even prior to the infant’s birth, as around 78% of CLP diagnoses are made during the 22 week prenatal ultrasound scan [43]. This diagnosis then instigates a series of actions that are designed to support the expectant mother and her unborn infant (e.g., provision of support materials containing images of cleft-affected infants, contact from a specialist cleft nurse [44]) but which also have a likely familiarising side effect on the mother. Mothers, upon receiving the diagnosis, also frequently report using internet search engines to view images of infants born with CLP [45]). This marks the beginning of a journey of discovery for the mother, that is likely to strengthen the process of familiarisation with infant CLP stimuli.

A previous study by the present authors investigated the role of prior familiarity with infant CLP on subjective “cuteness” ratings of cleft-affected infant faces and found participants who had previously been systematically familiarised with infant CLP to provide increased ratings of these infant faces following completion of a purpose-built familiarisation training phase [46]. In the current study, we have endeavoured to build on these findings by investigating the contribution of visual processing, by also measuring participants’ gaze behaviour and exploring its relationship with subjective appraisal of cleft-affected infant faces in the context of increased familiarity. As previously noted, the disruption to the baby schema brought about by the presence of the cleft is associated with an increase in visual attention to the mouth area and a decrease in "cuteness" ratings in naïve observers, with an inverse relationship between these two aspects of CLP-affected face processing (e.g., [15, 33]). Given that increased familiarity has been found to increase "cuteness" perception in previously naïve observers rating CLP-affected infant faces [46], it may be reasonable to expect increased familiarity to also be related to a decrease in visual attention to the mouth area, or at least an attenuation of the relationship between the intensity of this attention and the perception of "cuteness".

As in our previous study, we sought to recruit adult females, then to familiarise them with infant CLP stimuli. We elected to assess only female participants as studies that have investigated sex differences in the perception of infant faces have reported smaller effects, possibly due to the presence of males in the sample (because of the lower degree of variability in their responses, e.g., [12, 13, 17]), with the clearest results being found in studies using females only [47].

The current study explored the effect of familiarity on the subjective attractiveness ratings and on the proportion of time spent fixating on the mouth region (out of the total time spent gazing at the infant face) of both typical and atypical infant faces, among individuals who were actively familiarised with infant CLP stimuli, who were compared to a control group of naïve observers. In the experimental group only, a week-long active familiarisation training phase was undertaken prior to the eye tracking assessment and consisted of exposure to visual and informational material similar to that which mothers of infants with CLP receive prior to birth. Images of both cleft-affected, and typical infants were shown to both groups of female participants, who then provided ratings of “cuteness” of the target infant, while their eye gaze was measured using eye tracking. As previous research has found effects of both participant and infant age and infant gender on perception of infant faces [8, 10, 13], the effects of these variables were accounted for. Likewise, both gaze direction and head direction of target images have been found to be related to adult observers’ judgements of attractiveness [48, 49] and were also controlled for. Lastly, research has found participants display a tendency to work harder to view typically developing (TD) faces compared to those affected by a congenital facial malformation (including CLP [18]). We therefore measured and controlled for proportion of time viewing the face region of the target images (compared to outside the face region) during statistical analysis.

Our hypotheses were threefold. Firstly, we expected to find a replication of the findings from our previous study, in which familiarised participants provided higher “cuteness” ratings of CLP-affected infants compared to naïve participants [46]. Secondly, we hypothesised that, compared to naïve observers, familiarised participants would gaze for significantly less time at the mouth area of cleft-affected infant images. Lastly, in relation to cuteness ratings we expected to observe an interaction between presence of a cleft, experimental group, and gaze to the mouth area; specifically, it was predicted that, in control participants gazing at infant faces affected by CLP specifically (thus not for typically developing faces), a negative relationship would be found between cuteness ratings and percentage of time spent gazing at the mouth area, while no such relationship would be found in familiarised individuals.

## Method

### Participants

Eighty-four adult females aged 18–32 (M = 19.32±2.63) were recruited via opportunity sampling from the University of Liverpool undergraduate population and the general public. In order to be eligible, participants were required to be biologically female, not to be parents, to have normal or corrected to normal vision, and to have no prior familiarity with infants affected by CLP (which included not being a participant in our previous study on familiarisation with CLP). Participants were allocated at random into either the control (*n* = 41) or experimental groups (*n* = 43) using a random number generator. Experimental group participants received active familiarisation training prior to undertaking the eye tracking assessment (see S1 File for more detail re inclusion/ exclusion criteria). To determine the current study’s sample size, a power calculation was performed using the GLIMMPSE 3.0 Sample Size Software package [50]. The total suggested sample size was 76 participants for an effect size of f = .20 (statistical power being .95 with a significance value of .05). Therefore, a total sample size of 80 (40 per between-subjects group) was deemed sufficient for the present study, in anticipation of attrition. Participant recruitment took place between 20^th^ of February and 17^th^ of October 2022.

### Ethics statement

The research project was conducted in accordance with the ethical standards outlined by the Helsinki Declaration of 1975 [51], as revised in 2001 [52], and was approved by the University of Liverpool Institute of Population Health Research Ethics Committee (IPH-REC; project ID: 10011; July 2021). Written, informed consent was obtained from all participants before commencing the study.

### Materials

#### Infant face stimuli

**Fig 1:**
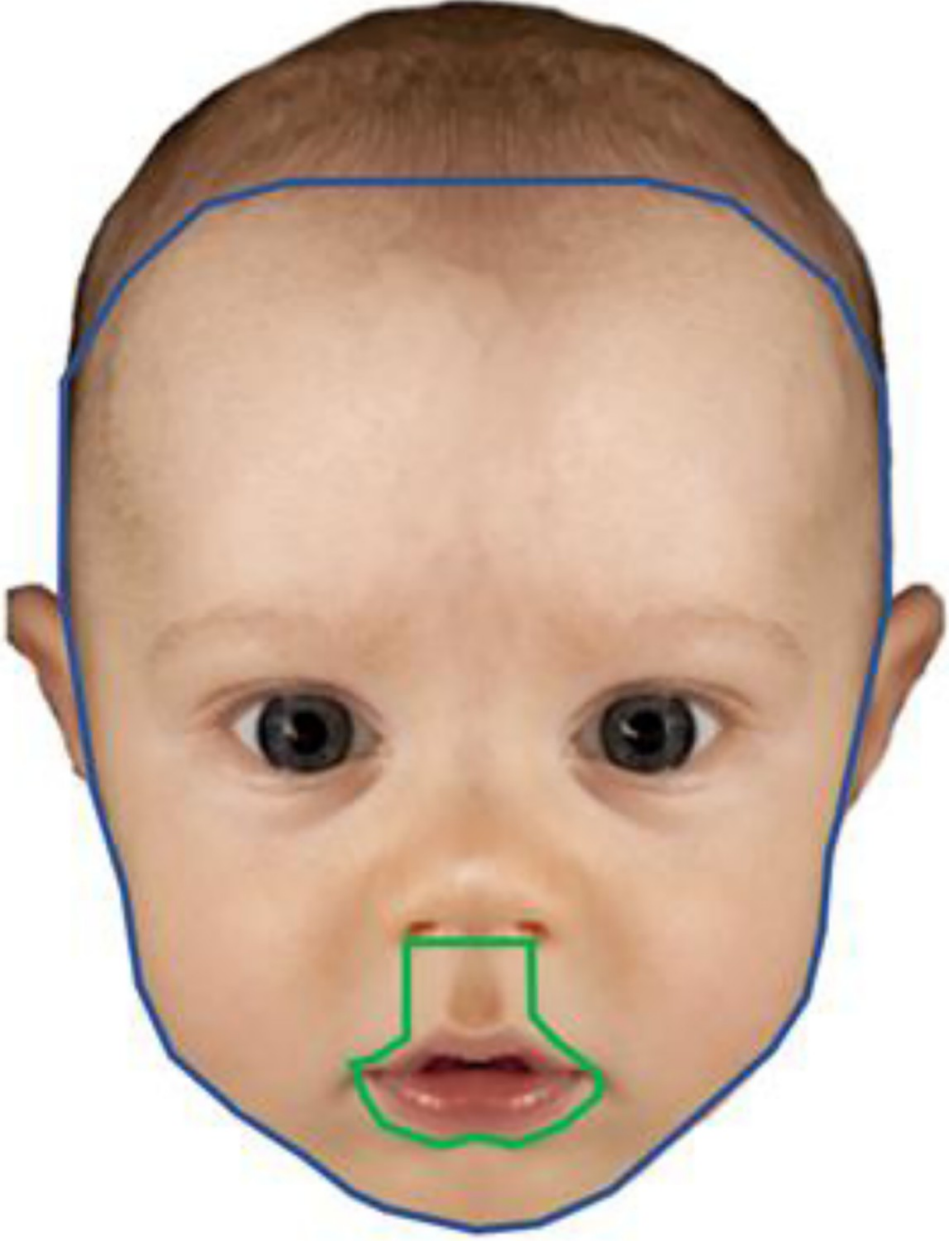
Example of a cleft-affected infant image with mouth and other facial interest areas. Image taken with permission from [14], adapted from [55].

Stimuli consisted of 96 images of infant faces (48 CLP) ranging in age from 3–13 (M = 7.79; sd 3.28) months. The stimuli were created using 48 images of typical infants, with a second version of each of these images being subjected to digital modification to make the infants appear to be affected by CLP [14]. See Hunt et al. [46] for a complete description of stimuli. The images were presented using SR Research Experiment Builder [53]. Areas of interest (AOIs) were created for each individual image in order to extract fixation location data from specific areas of the face. For the Mouth AOI, a polygon was drawn around the contours of the mouth, following its natural shape, ensuring complete inclusion of the mouth (the lower border was always below the bottom of the bottom lip). The upper border was extended to include the nostrils and included the upper lip in the form of a square or vertical rectangle (individual image dependent) as per Morzycki et al. [30], to capture fixations towards the upper lip. Finally, in defining the “Other facial areas” AOI, a final polygon was drawn following the natural shape of the face. The left-hand border included the left side of the face (but not the ear) up to the top left corner of the face (including forehead even if partially covered by hair). The same was true for the right-hand border. The lower border of the AOI was placed on or immediately below the chin, so that no white background was visible between chin and lower border. The AOI’s were also created using SR Research Data Viewer [54]. See Fig 1 for an example of AOI boundaries.

The 48 images of cleft-affected infants were rated on a scale of severity (mild, moderate, and severe) for a previous study (see Hunt et al. [46] for additional detail) by two independent raters who were blind to the aims of the study. Differences of opinion between the raters were resolved through discussion. Out of the three possible severity categories, seven images were rated as ‘mild’, 31 as ‘moderate’, and ten images as ‘severe.’

To account for their possible variability and effect on participant ratings, estimates of infant gaze and head direction were derived using the OpenFace Facial Behaviour Analysis Toolkit [56]. This toolkit provided eye gaze direction in radians, on both horizontal and vertical planes. Head direction estimates provided head rotation in radians around three possible planar axes, indicating head pitch, yaw, and roll. To minimise the number of potential predictors in the model, estimates of general gaze and head off-centeredness were derived through summation of the absolute values of OpenFace estimates for gaze direction, and for head direction, respectively, with zero representing perfect centeredness.

#### Infant attractiveness ratings

Participants were instructed to provide “Cuteness” ratings for each image that was presented, adapted from a study conducted by Lewis et al. [14]. The Likert scale was presented following each infant image, with participants required to select a value ranging 1–7 in response to the questions “How cute do you rate this infant?” (“Not cute at all” to “Extremely cute”) using a mouse click on a sliding scale. The question was presented on a subsequent screen with the sliding scale situated immediately beneath the question. Upon completion of the Likert response, the screen automatically advanced to the next infant image. The questions were also presented in SR Research Experiment Builder [53].

#### Active familiarisation (AF) training development

The AF training materials were created using informational and audio-visual material on infant CLP, including the use of content that was more condensed but similar in nature to what pregnant mothers, whose unborn infant is diagnosed with CLP, seek and receive. These materials were created using informational literature that is provided to mothers upon diagnosis of the cleft (either at 22-week scan or upon delivery of infant) such as educational leaflets created by Northwest England, The Isle of Man, and North Wales Cleft lip and Palate Network [44], and public health information websites such as the NHS [57]. Participants were required to access seven daily instalments comprising approximately 20 slides per session. A typical slide comprised text of around 50 words, accompanied by an image of an infant born with CLP that took up approximately half the size of the entire slide. Most images were of the infant pre-surgery, however, where information was given about infants post-surgery, a small number (<11%) were images of children post-surgical procedure. Each daily instalment featured a comprehension check question that had to be answered correctly in order for participants to proceed. This ensured participants were reading and comprehending the information. Materials were developed and presented using the Gorilla Experiment Builder (Gorilla.SC). See Hunt et al. [46] for a complete description of training materials.

### Procedure

Participants were identified and recruited largely through the use of a university-based system designed to promote research participation among its undergraduate students. A small number of non-students (*n* = 9) responded to an advertisement posted on University premises. Once individuals had indicated their willingness to participate, participants were allocated into either the control group or AF group at random. Control participants were invited to undertake the eye tracking assessment at their convenience and were provided with participant information and consent forms prior to testing. AF participants received an email invitation that included a hyperlink and a unique password that enabled access to the AF training. Participants were informed that engagement would be monitored throughout the seven daily training sessions via a checkpoint system (function settings within Gorilla.sc allow experimenters to determine the amount of time spent on each instalment and to be notified once each is completed). This enabled the research team to ensure that participants were being exposed to the training materials. Upon completion of the training, AF participants were invited to undergo the eye tracking assessment, as soon as was practicably possible.

All participants were subjected to the eye tracking assessment, which comprised a single block of 48 trials (24 TD images, 24 CLP images) with one trial consisting of viewing a single image for ten seconds and providing a subjective rating of “cuteness” for each infant image. Images were shown at random, and participants saw each image only once. Two versions of the task were created so that no participant viewed an image of a typical infant that was also affected by a cleft (or vice versa). Eye tracking was performed using the EyeLink 1000 plus LCD arm mount eye tracker [53]. Data was obtained using monocular left eye tracking with a 500Hz sampling rate. Images were presented on a screen with 1024*768 resolution (4:3 ratio). Participants were seated at a table 55cm from the display screen. A chinrest was used to reduce head movement and testing took place in an enclosed space to minimise distraction. A standard 9-point calibration system was used to calibrate the eye tracker prior to testing. A drift check was performed after each trial to monitor tracking accuracy. This required participants to focus on a central fixation cross in between trials to ensure gaze position was maintaining < 2° of visual angle from the target. Participants’ eye gaze was monitored throughout the duration of the assessment, however, only data that was collected during the ten second ‘image’ phase of each trial was retained. All eye tracking data collected during the rating scales phase of the trials was disregarded. All trials with less than five total seconds of valid gaze data were excluded prior to analysis. Data was extracted using SR Research Data Viewer [54]. Participants were informed that their responses were completely anonymous. Upon completion of the final trial, participants were debriefed and given the opportunity to ask questions (see S1 File for a flow chart of the study procedure).

### Analysis strategy

Overall percentages of fixations to the two AOIs over time were extracted using Dataviewer [54]. A recent review of eye tracking to cleft-affected faces studies [58] recommended the use of brief trial times (e.g., shorter than five seconds) due to practical issues surrounding participant engagement and fatigue. We therefore visually explored a detailed time-based representation of the data (obtained by averaging the likelihood of mouth-fixation every 2ms over the course of each 10s trial, per group and face type), in order to determine the optimal range of data to retain for study purposes, between when participants started reacting to the visual stimuli and before participant fatigue manifested itself, over the course of the original ten-second trials. Following this visual inspection of the data, the optimal window in which to perform inferential analyses on was deemed to lie between 250ms and 1750ms for each 10s trial (see S1 File).

To investigate the differences between groups, two linear mixed models (LMM) were performed. Both LMMs contained fixed effects of cleft (CLP vs. TD), and group (Familiarised vs. Control), and their interaction. Random effects of Participants and Image were included on the intercept. Variables controlled for in both models were participant age, infant age and gender, severity of cleft, and both head and eye gaze direction of the target images, due to the previously described possible effects on perception of infant faces. Lastly, trial number was included as a covariate in anticipation of variable participant engagement during the early versus later stages of the task.

The first model was created in order to test whether familiarised participants would gaze for significantly less time at the mouth area of cleft-affected images compared to control participants. Thus, this model employed Fixation duration (average proportion of fixation time to mouth area versus the rest of the face [as a percentage] for each trial) as the dependent variable.

The second model was intended to test whether familiarised participants would provide higher “cuteness” ratings of cleft-affected infants, and whether there would be an interaction between presence of the cleft (CLP versus TD), and participant group, and whether the effect of this would change with varying gaze to mouth. For this model, “cuteness” rating was employed as the dependent variable and fixation duration to mouth was included as a fixed effect, in interaction with group and cleft.

Multiple comparisons used False Discovery Rate correction [59]. Restricted maximum likelihood was applied to the estimated models, and statistical values for individual model effects were obtained using the mixed() function of the “afex” package in R version 4.2.2 [60, 61], which obtained Type 3 tests by comparing models in which only the tested effect was excluded against the full model (i.e., including all effects). Degrees of freedom were computed using the Satterthwaite method. Detailed results (e.g., parameter estimates) are reported in tabulated format in line with recommendations made by Meteyard and Davies [62] and can be found in the S1 File.

## Results

### Descriptive statistics

For the experimental group, both time spent viewing the AF training materials overall, and number of days taken to complete the training, was in line with the sample in our previous study (Hunt et al., 2023). See Table 1 for descriptive statistics and information regarding AF training exposure.

**Table 1:** Summary statistics for the familiarised and control groups. Values are Mean (sd).

	Familiarised group	Control group
Age	19.53 (2.99)	19.10 (2.17)
Total time spent on AF materials (minutes)	43.75 (18.06)	-
Number of days taken to complete the training	8.74 (2.71)	-
Number of days between completion of training and undertaking the assessment	4.40 (3.56)	-

Broadly, when comparing within the groups, CLP images were provided with lower “Cuteness” ratings, and received greater Gaze time to mouth region, compared to typical images. See Table 2 for descriptive statistics.

**Table 2:** Descriptive statistics for “cuteness” ratings and gaze time to mouth area, according to experimental group and image type. Values are Mean(sd).

	Familiarised group		Control group	
	TD images	CLP images	TD images	CLP images
“Cuteness” ratings	4.79 (1.41)	4.68 (1.35)	4.30 (1.39)	4.21 (1.29)
Proportion of gaze time to mouth area (% of trial)	19.07 (18.87)	26.81 (22.26)	16.53 (17.41)	23.33 (21.57)

#### Model 1. Fixation duration to mouth area

For the first tested LMM, the conditional R squared value was 0.39 (38.6% of variance explained by whole model) and the marginal R squared value was 0.05 (5.3% of variance explained by fixed effects only). Addressing the a priori hypotheses, with regards to fixation to mouth area, no Group * Cleft interaction was found (F(1, 3888.38) = 0.10, p = .757). AF and control participants did not differ in the extent to which they fixated on the mouth area of either CLP images or TD images. Proportion of gaze time to mouth area (% of trial), split by Group and Image type, is provided in Table 2.

There were several notable secondary findings from model 1. With regards to fixation to mouth region, an effect of Cleft was observed (F(1, 3877.90) = 194.44, p < .001). Regardless of experimental group, proportion of viewing time was significantly higher towards the mouth region of cleft-affected images (estimated M = 24.97; SE = 1.41) compared to typical images (estimated M = 17.81; SE = 1.41).

There was also an effect of Trial (F(1, 3890.92) = 54.22, p < .001). Fixation duration to mouth region was greater for images seen earlier in the block of 48 trials (b = -0.14, SE = 0.02, p < .001). Lastly, there was an effect of time spent gazing within the boundary of the face (F(1, 3946.69) = 20.89, p < .001), with a positive relationship emerging between this and gaze to mouth region (b = 0.18, SE = 0.04, p < .001), such that, the more time spent gazing at the target face stimulus, the more of this time was spent gazing at the mouth AOI.

#### Model 2. Fixation duration to mouth area and “cuteness” ratings

For the second tested LMM, the conditional R squared value was 0.52 (52% of variance explained by whole model) and the marginal R squared value was 0.09 (9.1% of variance explained by fixed effects only).

With regards to the third hypothesis, a Fixation * Group * Cleft interaction was found (F(1, 3890.73) = 6.97, p = .008). Probing the coefficients within this model showed a significant, negative relationship between “Cuteness” ratings and Gaze to mouth region for naïve participants viewing CLP images (b = -0.14; SE = 0.03; p < .001) but not typical images (b = 0.05; SE = 0.04; p = .195), with the former relationship being significantly more negative than the latter (b = -0.19; SE = 0.05; p < .001). No relationship was found between Cuteness ratings and Gaze to mouth area for AF participants viewing either typical images (b = -0.03; SE = 0.04; p = .449) or CLP images (b = -0.05; SE = 0.03; p = .127), with no difference between the relationships for these two kinds of faces in this group (b = -0.02; SE = 0.04; p = .650). Lastly, and notably, for typical images, the relationship between “Cuteness” ratings and Gaze to mouth region was found not to differ between control and AF participants (b = 0.08; SE = 0.06; p = .137), while the former showed a significantly more negative relationship between these two variables, compared to the latter, for CLP images (b = -0.09; SE = 0.05; p = .044). See Table 2 for descriptive statistics of Gaze to mouth region and “Cuteness” ratings, split by Group and Image type.

Regarding the first hypothesis, no Group * Cleft interaction was found from this model (F(1, 3811.21) = 0.00, p = .951). However, there were a number of notable secondary findings from this model. An effect of Group was observed. (F[1, 81.19] = 6.44, p = .013). Familiarised participants (M = 4.75; SE = .14) provided higher “cuteness” ratings to images overall compared to control participants (M = 4.28; SE = .14).

There was also an effect of Cleft (F[1, 3879.96] = 7.88, p = .005). Regardless of Group, TD images (M = 4.56; SE = .11) received higher “Cuteness” ratings compared to CLP images (M = 4.47; SE = .11).

There was also a significant effect of infant age (F(1, 43.01) = 4.73, p = .035), with younger infants receiving higher Cuteness ratings (b = -0.04, SE = 0.02). Significant effects of both Gaze direction and Head direction of infant images were observed, with higher ratings provided for images where the gaze (eye) direction (F(1, 42.98) = 17.61, p < .001, b = 5.31, SE = 1.26), and head direction (F(1, 43.01) = 5.92, p = .019, b = -2.65, SE = 1.09) of infant images were more off-centre. Finally, there was an effect of Trial (F(1, 3880.76) = 41.39, p < .001, b = -0.01, SE = 0.001), where images seen earlier in the block of 48 trials received higher Cuteness ratings.

## Discussion

We investigated the role of systematic familiarisation in adult females’ perception of infant faces–both with and without CLP–by measuring their eye gaze behaviour and subjective appraisal (“cuteness” ratings) towards both types of target infants. Crucially, we also investigated the possible influence familiarisation may have on the relationship between fixation duration to mouth area of the cleft-affected infant images and the “cuteness” ratings they subsequently received.

Firstly, it was expected that familiarised participants would provide higher “cuteness” ratings of cleft-affected infants compared to naïve participants. Secondly, it was predicted that familiarised participants would gaze for significantly less time at the CLP affected infants’ mouth area. Lastly, we expected to observe a relationship between gaze behaviour and subjective ratings, varying according to familiarisation and CLP presence, such that a significant negative relationship would emerge between cuteness ratings and gaze to mouth region for control participants when viewing images of CLP affected infants specifically, but that this relationship would not be present for familiarised participants.

In respect of the first hypothesis, familiarised participants were not found to provide higher “cuteness” ratings of CLP-affected infants compared to naïve participants. This was surprising, given that, in our previous study, participants who had undertaken the familiarisation training found cleft-affected infants “cuter” than naïve participants, who received no training [46]. The overall pattern of results from the present study resemble those reported in our previous study, albeit the group difference in “cuteness” ratings was not statistically significant.

The most prominent differences between these two studies are methodological in nature. It may be that, in the present study, the inclusion of an eye tracking measure played a role in attenuating the difference in ratings between the groups. Participants’ awareness of the eye tracking component of the study may have subtly altered their visual scanning patterns, and subsequently, their processing of the stimuli, bringing to the fore the visual characteristics of the cleft and narrowing the effect of group on “cuteness” ratings. Secondly, in our previous study, the experimental task was conducted online, with participants likely to be undertaking the “cuteness” ratings task at home, whereas, in the present study, the ratings task was conducted in the lab. Given the presence of the researcher in the present study, the effect of social desirability, especially for those participants who had been familiarised, may have been stronger than for those participants who did everything in the comfort and privacy of their homes. This may have contributed to the generally higher “cuteness” ratings in the familiarised group in the present study, and this overall increase may have impinged on the specific effect on cleft-affected stimuli, thereby reducing the likelihood of finding a significant interaction between group and cleft. The role of the inclusion of eye tracking, and its subsequent effect on “cuteness” ratings may be an avenue for future research.

With regards to the second hypothesis, gaze duration to mouth area of CLP affected images did not differ between familiarised and control participants, which was unexpected, given individuals highly familiar with CLP stimuli (e.g., mothers of cleft-affected infants) have been found to gaze away from the cleft-affected infant’s mouth area [28].

One possible explanation for this unexpected result is related to the degree of exposure that familiarised participants experienced. As participants were subjected to an average of just under 44 minutes of training over the course of a single week, it is possible that this amount of exposure to infant CLP stimuli is not sufficient to influence participants’ gaze patterns alone. De Pascalis et al. [28] focused on the first two months of infant life, meaning the mothers included in the study had a number of weeks of exposure to their infants’ clefts. As discussed earlier, these mothers may also have been familiarising themselves with infant CLP from before the birth, if their infant had been diagnosed with a cleft during the 22-week scan. This level of familiarity, therefore, substantially outweighs that of the AF participants in the present study and may explain why the AF training did not influence participants’ gaze behaviour in isolation.

Naturally, it is likely that the differences between these studies are also driven by the parental role exclusive to the participants in the study by De Pascalis and colleagues [28], and the potential negative emotional correlates of the birth of an infant with CLP. Research on parental adjustment to congenital disfigurement has found that mothers can experience grief and shock upon learning of their child’s malformation, with one mother reporting that she “didn’t want to look” at her cleft-affected infant’s face, choosing instead to look at the infant’s feet [63]. Investigation of these two factors within the same study may be an avenue for future research, as it is plausible that these factors play a role in face perception of one’s own infant. A further factor playing a role in this difference between studies, and one that should also be investigated in future research, is tied to the nature of the infant stimuli used. While De Pascalis and colleagues [28] investigated naturalistic interactions, we presented static images of infants in an environment where participants were free to gaze towards the images without fear of causing offence or flouting social norms (e.g., staring unapologetically at the malformed face of another person). Langer et al. [64] found increased fixation towards atypical characteristics in participants viewing still images of individuals with a disability, but that this tendency to fixate decreased when the viewer was aware that they are being observed by a third party.

There were also several notable secondary findings from the model used to test the first hypothesis. We observed a significant effect of cleft on fixation duration to mouth region. Regardless of experimental group, participants fixated significantly more towards the mouth region of cleft-affected images compared to typical infants, a finding that is consistently reported in the literature on visual processing of cleft-affected faces [58] for targets of both infant (e.g., [15, 16], child [30], and adult [31, 33, 65] age.

Regarding the third hypothesis, a significant, negative relationship was found between gaze to the mouth region and “cuteness” ratings of CLP affected infants, for control participants, as predicted. Further, the relationship between gaze and “cuteness” ratings towards CLP images was found to be significantly more negative than the relationship between these variables for control participants viewing TD images (where this relationship was not found to be significant). No relationship between gaze to the mouth region and “cuteness” ratings was found for familiarised participants viewing either image type, and no difference between the two kinds of pictures was found in these participants, regarding this relationship.

Our finding is consistent with those reported by Boonipat et al. [33] who found decreased fixation time to the cleft-affected lip region for infants who were rated as more attractive. Similarly, Rayson et al. [15] reported a positive relationship between “cuteness” ratings and gaze to eye region (at the expense of gaze to the mouth) for cleft-affected infants. These findings, alongside our own, indicate that the less attractive the cleft-affected infant is perceived as, the greater the degree of gaze to the affected mouth area is likely to be.

This finding may not be entirely surprising, given the widely reported aversion to the cleft-affected infant face often found among naïve participants in experimental studies (e.g., lower ratings of hypothetical caregiving compared to TD infants [14, 15]), possibly due to evolutionary facial cues pointing to poor genetic fitness [66].

Nevertheless, notably, AF training appeared to abolish this effect in the familiarised participants, with the AF training possibly having an effect of increased acceptance of infant CLP stimuli in the participants who engaged in it, even in the context of non-decreased viewing time to the cleft-affected area. Interestingly, this effect seemed to only be abolished for CLP stimuli, with no difference found between groups in the relationship between gaze and ratings for TD stimuli, indicating how the effect of familiarisation specifically affected CLP processing, rather than infant-related reactions generally. While this pattern of findings appears to be highly suggestive, future studies may obtain further clarity by contrasting the familiarisation training with a similar (in duration and structure) task for control participants (e.g., familiarisation with visual and informational content related to typical infants).

The reported results are encouraging; caution should however be exercised when interpreting eye gaze behaviour as being directly related to aesthetic preferences. One study that explored the relationship between aesthetic ratings and gaze fixations towards varying lip volumes (increased/ decreased size) and proportions (upper to lower lip ratio) found that the lip volume that received the highest ratings was not the target that induced the longest fixation times. A similar effect was found for lip proportion. Broadly, this finding suggests that participants do not necessarily fixate for longest on the target that they ultimately find more or less attractive.

Regarding “cuteness” ratings in isolation, a significant effect of Group was found. Specifically, AF participants provided higher “cuteness” ratings overall compared to control participants. This finding is perhaps in keeping with the effect of “mere exposure” whereby participants with prior experience of a stimulus show a discernible “liking” effect on second presentation of a target [67].

There was also an overall effect of Cleft on “cuteness” ratings. In keeping with previous research [14–16], typical infants received higher “cuteness” ratings compared to CLP infants, when ratings were viewed in isolation (i.e., when eye gaze data was not a factor).

We found a significant effect of infant age, such that, younger infants received higher “cuteness” ratings. This finding is consistent with those reported in our previous study [46], in which younger infants received higher “cuteness” ratings. Similarly, Luo et al. [2] found younger children were judged as more likeable and attractive than older children. That younger infants might be considered “cuter” or more attractive than older infants perhaps speaks to the evolutionary role of Kindchenschema in adult-infant caretaking behaviour, in that, clear signals of youth and vulnerability are met with interest in caregiving on the part of the adult.

Both gaze and head direction of the target infant images were found to be significantly associated with “cuteness” ratings, with higher ratings provided for images where both gaze and head direction were more off centre. Regarding head direction, this result is consistent with that reported in a previous study by this research team [46] which also found higher “cuteness” ratings were provided for infant images where the head direction was further off centre.

Regarding eye direction, the direction of this finding contrasts with those reported in studies assessing responses to infant [46] and adult faces [48], as participants in these studies gave higher ratings to images where gaze direction was nearer to centre. No specific hypotheses were made in regard to these findings, however, these results, and their inconsistency with findings from studies using both infant and adult facial stimuli suggests future research may wish to explore the role of eye and head direction of stimuli and their role in appraisal of infant faces during visual processing.

Lastly, we observed a significant effect on both fixation duration and “cuteness” ratings of trial number. Fixation duration to mouth region was greater for images seen earlier in the block of 48 trials, and “cuteness” ratings were higher for these images. That participants fixated less on the mouth region and provided lower “cuteness” ratings for images seen later in the block of 48 trials may have been due to progressive disengagement from the task. Respondent fatigue–defined as diminished response quality in the latter sections of a task–is a well-established phenomenon in psychometrics (e.g., survey completion) [68], which perhaps explains the effect of reduced subjective ratings towards the latter stages of the block of trials. However, our result suggests this effect of progressive disengagement may have also occurred for participants’ eye gaze behaviour, characterised by a progressive decrease in visual scanning of the mouth region, perhaps as a result of the length of the task. Our finding might therefore encourage future research to control for this variable in statistical analyses.

The design of the stimulus presentation was based on a study by Rayson et al. [15], who opted to collect eye gaze data towards target infant images for 10 seconds per image. After performing a visual inspection of the data, we opted to conduct statistical analyses on the 1500ms window in which the largest variations in fixation to mouth region appeared in both groups, which reduced the noise present in the data. Other, similar studies also opted for shorter stimulus presentation periods, such as 4 seconds [5], 5 seconds [31], and 6 seconds [29]. Shorter stimulus presentation periods also reduce the overall testing time for the participant, and might, to some extent, minimise the aforementioned effect of trial number, in which participants appear to become less engaged with the task the further into it they progress.

We have built on our previous study on familiarity with infant CLP stimuli and attractiveness ratings and included an additional, instrumental measure of processing of infant CLP, specifically, the inclusion of an eye tracking measure, which represents a strength of the present work. As in our previous study, we measured the head and gaze direction of the face stimuli, as previous research has found that both head and gaze direction of target face stimuli are related to subjective perceived attractiveness among observers [48, 49]. We therefore elected to control for these within the statistical analysis, as per Rayson et al. [15].

The study did have a number of limitations. The AF training was participant initiated, in that, individuals who had been allocated into this experimental group were free to access the training materials when it was convenient for them to do so. This led to variation in several time-related variables (number of days from completion of AF training to lab assessment, number of days taken to complete all seven instalments of the training, total time spent on all seven instalments). Future work may choose to enact a greater degree of control of these variables, for example, by conducting the training in a lab environment where participants’ engagement with the training materials can be more closely monitored.

A further limitation relates to the design of the study. As the present study adopted a correlational design, it was not possible to infer a causal relationship between the AF training and the outcomes (“cuteness” ratings, eye gaze behaviour) from the present data. Although study design is not the only prerequisite for a high quality randomised controlled trial capable of demonstrating causality (e.g., use of a parallel training arm for the control group), future research may wish to employ a pre-test-post-test design to investigate a potential causal relationship between prior familiarity and responses to CLP-affected infant faces.

A final limitation of the present work was the use of only two outcome variables (responses to “cuteness” ratings and fixation to mouth area). The use of additional outcome variables has the potential to increase confidence when drawing conclusions from the findings. Future research may wish to explore whether participants’ empathic attitudes can be strengthened, or whether perspective taking (e.g., through the use of vignettes) is associated with the perception of cleft-affected infants.

One implication of the findings from the present work is that although the effect of the AF training on face processing could be detected, the training load that participants were subjected to was not sufficient to influence gaze patterns alone when viewing infant images affected by CLP. It may be the case that the degree of exposure that participants experienced during the training, whether its length or strength, lacked the behaviour modifying qualities required to influence gaze patterns to the mouth region of cleft-affected infant faces alone. Therefore, differing degrees of exposure to AF training may be an avenue for future research.

A further implication is related to modification of the perceiver’s experienced prior to viewing CLP-affected infants. Whilst previous studies measuring adult ratings of infant faces have tended to explore these effects via manipulation of facial features of the input stimuli [5, 6], the present study is one of a few that have shown how adult evaluations and reactions to infant faces can be modified by manipulating the participants’ experience [47, 69].

We have demonstrated that it may be possible to attenuate aversive responses to individuals with congenital facial malformations, which has implications for how these individuals are treated in social situations. Given children born with CLP may experience teasing and bullying while at school due to their appearance [70], our findings suggest it may be possible to increase its level of acceptance through systematic prior familiarisation with the malformation in question among the affected child’s peers, and with regards to the broader social environment, it may be possible to increase positive public perception of infant CLP.

In conclusion, we investigated the role of familiarity and its effect on eye gaze behaviour and subjective ratings towards cleft-affected and typical infants in two groups of females: naïve participants and familiarised participants. This latter group completed a novel, purpose-built seven-day familiarisation training programme prior to the eye gaze and ratings assessments, while naïve participants were not subjected to any intervention. We found a significant negative relationship between “cuteness” ratings and gaze time to the mouth area for cleft-affected infant images for control participants only, such that, the greater the time spent gazing at the mouth region, the lower the subjective ratings were found to be. This effect was not observed for participants who had undertaken the familiarisation training. Our findings suggest familiarity with infant CLP has the potential to provide an ameliorating effect on individuals’ typically observed aversive responses to cleft-affected infant faces.

## Supporting information

S1 File Supporting information figure should appear here. (DOCX)
