# Supplementary material for: In the eye and mind of the beholder: The effects of familiarisation on the perception of atypical infant facial configurations
Source: PLoS One. 2024 Nov 12;19(11):e0311763. doi: 10.1371/journal.pone.0311763 (PMC11556683; doi:10.1371/journal.pone.0311763)
Supplement: S1 File — (DOCX) [file pone.0311763.s001.docx]

Supplementary materials

**Supplementary Figure S1. Gaze to mouth area (likelihood percentage) for familiarised and control participants, split by image type.**

The shaded area shows the time interval selected for data analysis in the present study.


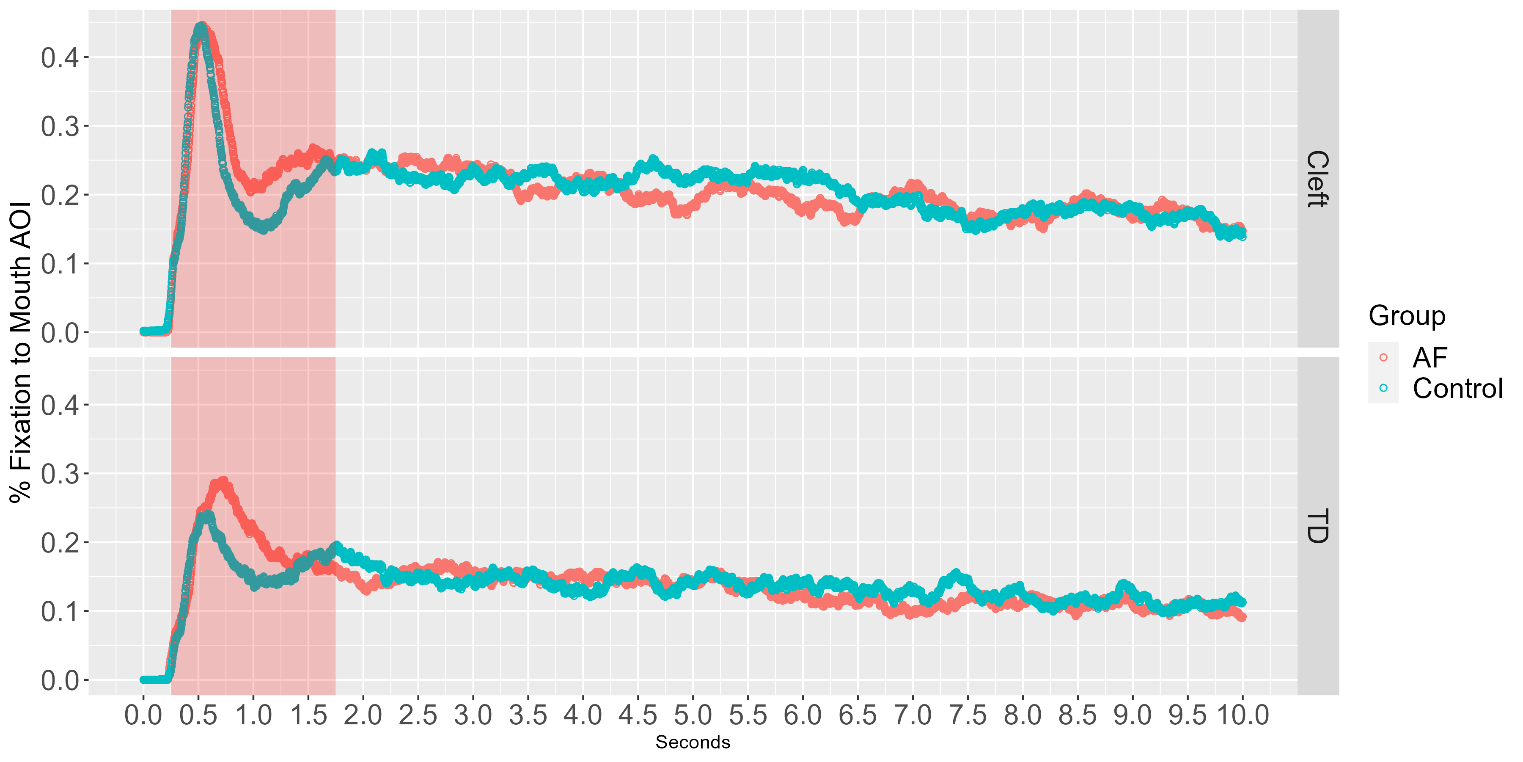


**Supplementary figure S2. Flow chart of recruitment procedure.**


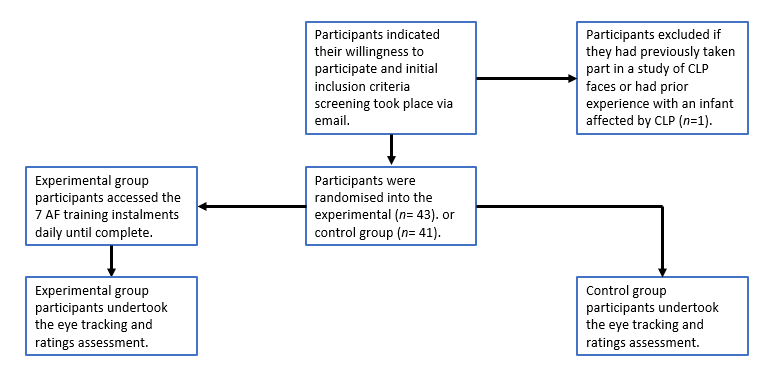


**Supplementary table 1. Coefficients for fixation duration to mouth area (Model 1).**

|  | B | SE | 95%CI | t | p |
| --- | --- | --- | --- | --- | --- |
| Intercept | -0.67 | 10.50 | -21.13 – 19.64 | -0.06 | .949 |
| Group | 1.57 | 1.26 | -0.89 – 4.04 | 1.24 | .218 |
| Cleft | 3.58 | 0.26 | 3.07 – 4.08 | 13.94 | <.001 |
| Trial Number | -0.14 | 0.02 | -0.17 – -0.10 | -7.36 | <.001 |
| Age | 0.11 | 0.48 | -0.83 – 1.04 | 0.22 | .826 |
| Face Fixation | 0.18 | 0.04 | 0.10 – 0.26 | 4.57 | <.001 |
| Infant age | 0.17 | 0.19 | -0.20 – 0.53 | 0.85 | .398 |
| Infant gender | -0.14 | 0.63 | -1.35 – 1.07 | -0.22 | .830 |
| Gaze direction | 5.54 | 12.72 | -18.73 – 29.82 | 0.44 | .665 |
| Head direction | 13.07 | 10.94 | -7.80 – 33.95 | 1.20 | .238 |
| Group * Cleft interaction | 0.08 | 0.26 | -0.42 - 0.59 | 0.31 | .757 |

**Supplementary table 2. Coefficients for fixation duration to mouth area and “cuteness” ratings (Model 2).**

|  | B | SE | 95%CI | t | p |
| --- | --- | --- | --- | --- | --- |
| Intercept | 4.16 | 0.77 | 2.67 – 5.65 | 5.42 | <.001 |
| Group | 0.23 | 0.09 | 0.41 – 0.05 | 2.54 | .013 |
| Cleft | -0.04 | 0.02 | -0.08 – -0.01 | -2.81 | .005 |
| Fixation to mouth | -0.002 | 0.001 | -0.004 – -0.0001 | -2.05 | .04 |
| Trial | -0.01 | 0.001 | -0.01 – -0.01 | -6.43 | <.001 |
| Age | 0.04 | 0.03 | -0.03 – 0.11 | 1.19 | .237 |
| Infant age | -0.04 | 0.02 | -0.08 – -0.01 | -2.18 | .035 |
| Infant gender | 0.12 | 0.06 | -0.003 - 0.24 | 1.86 | .071 |
| Face fixation | 0.002 | 0.002 | -0.02 – -0.01 | 1.04 | .297 |
| Gaze direction | 5.31 | 1.26 | 2.90 – 7.72 | 4.20 | <.001 |
| Head direction | -2.65 | 1.09 | -4.72 – -0.57 | -2.43 | .019 |
| Group * Cleft interaction | 0.001 | 0.02 | -0.03 – 0.03 | -0.06 | .951 |
| Group * Fixation to mouth interaction | -0.0001 | 0.001 | -0.002 – 0.002 | -0.12 | .902 |
| Cleft * Fixation to mouth interaction | -0.003 | 0.001 | -0.004 – -0.001 | -3.25 | .001 |
| Group * Cleft * Fixation to mouth interaction | -0.001 | 0.02 | 0.004 - 0.001 | -2.64 | .008 |
